# Safety of concurrent treatment of cats with fluralaner and emodepsid–praziquantel

**DOI:** 10.1186/s13071-016-1618-y

**Published:** 2016-06-06

**Authors:** Feli M. Walther, Mark J. Allan, Rainer K. A. Roepke

**Affiliations:** Merck Animal Health, 2 Giralda Farms, Madison, NJ USA; MSD Animal Health Innovation GmbH, Zur Propstei, 55270 Schwabenheim, Germany

**Keywords:** Cat, Fluralaner, Bravecto™, Emodepsid, Praziquantel, Profender™, Safety

## Abstract

**Background:**

Fluralaner is a novel systemic ectoparasiticide for cats providing immediate and persistent flea- and tick-control after a single topical dose. Emodepsid and praziquantel are routinely used to control intestinal worm infections in cats. The safety of concurrent use of fluralaner and a commercially available emodepsid-praziquantel combination topical solution was investigated using topical administrations at the maximum recommended dose rates.

**Findings:**

Few mild and transient clinical findings like erythema at the administration site and single incidences of salivation or vomiting were observed. All of which were consistent with the individual product leaflets. There were no findings suggesting an increased safety risk associated with the concurrent treatment of cats with fluralaner and emodepsid-praziquantel.

**Conclusions:**

Concurrent treatment with fluralaner, emodepsid and praziquantel is well tolerated in cats.

## Findings

Fluralaner is a novel isoxazoline class compound that provides fast and persistent insecticidal and acaricidal efficacy in cats [1]. Following topical administration fluralaner is absorbed transdermally [2] providing at least 12 weeks of flea- and tick-control (*Ctenocephalides felis, Ixodes ricinus*) after a single dose [1]. Fluralaner was shown to be safe when administered topically at overdoses of up to 5 times the maximum clinical dose, i.e. up to 465 mg fluralaner/kg body weight, at 8-week intervals in healthy cats [1] and when administered off label orally, to mimic accidental lick-off following topical administration, at the maximum clinical dose of 93 mg fluralaner/kg body weight [1]. Emodepsid is active against larval and adult stages of intestinal nematodes, and praziquantel is active against cestodes in cats [3].

Cats may concurrently be infested with ticks, fleas and intestinal helminths, e.g. with *Dipylidium caninum* (flea tapeworm) transmitted during flea infestation. Therefore, veterinarians may choose to administer an ectoparasiticide like fluralaner concurrently with endoparasiticides such as emodepsid and praziquantel. Fluralaner (Bravecto Spot-on solution™; MSD Animal Health) and emodepsid/praziquantel (Profender™; Bayer Animal Health GmbH) are topical formulations with transdermal absorption following administration. Both products may potentially be associated with mild and transient adverse events following treatment, as specified on the products’ labels. These include salivation and vomiting, potentially a result of the cat licking the application site immediately after treatment, or erythema, alopecia and pruritus at the topical administration site [1, 3].

This study evaluated the safety of concurrent use of fluralaner and an emodepsid-praziquantel combination in healthy cats at the recommended treatment doses.

Twenty healthy European mixed breed cats (ten males and ten females) were included in the study.

Twenty cats 1.3–6.6 (mean 2.5) years of age and weighing 2.1–6.4 (mean 3.9) kg were allocated to two study groups by sorting within gender according to descending body weight and then random allocation to a group. Cats of the control group were topically administered emodepsid-praziquantel at a dose of 6.25 mg/25 mg/kg body weight on six occasions at 4-week intervals. Cats of the treatment group also received emodepsid-praziquantel at the same dose and schedule, together with fluralaner at 93 mg/kg body weight on three occasions at 8-week intervals, i.e. immediately after the first, third and fifth emodepsid-praziquantel administration (Table 1). The emodepsid-praziquantel solution was administered at the base of the skull, and fluralaner was administered caudal thereof on the three days when both products were administered concurrently (days 0, 56, 112).

**Table 1:** Study group details for evaluation of the safety of fluralaner and emodepsid-praziquantel when administered concurrently to cats

		Control group	Treatment group
Emodepsid-praziquantel treatment days		0, 28, 56, 84, 112, 140	0, 28, 56, 84, 112, 140
Fluralaner treatment days		–	0, 56, 112
Emodepsid dose (mg/kg body weight)		6.25	6.25
Praziquantel dose (mg/kg body weight)		25	25
Fluralaner dose (mg/kg body weight)		–	93
Gender	Male	5	5
	Female	5	5
Body weight (kg) on study day -1	Mean ± SD	4.0 ± 1.4	3.8 ± 1.3
Body weight (kg) on study day 168	Mean ± SD	4.1 ± 1.4	4.0 ± 1.5

*SD* standard deviation

Cats of both groups were observed for general health twice daily throughout the study and body weights were recorded weekly. All cats were examined by a veterinarian on the day prior to each topical treatment, 16 days following each topical treatment and at the end of the study (day 167). Examinations included assessments for abnormalities in behavior, locomotion, musculoskeletal system, coat, skin, superficial lymph nodes, eyes, pupils, ears, oral cavity, mucous membranes, capillary refill time, respiration, auscultation of heart and respiratory tract, heart rate, respiratory rate, pulse, abdominal palpation, rectal temperature, any other visible abnormalities and body condition. In addition, all cats were observed for general health during the first hour following each treatment, and were assessed by a veterinarian prior to each treatment and 1, 2, 4, 6, 12, 24, 36, 48, 60, 72, 96, 120, 144 and 168 h following each treatment for abnormalities in behavior, locomotion, respiration, cardiovascular system, eyes/pupils, coat and site of application, pruritus, salivation and cage side findings.

Throughout the 24-week study period, there were no findings that were considered to be related to the concurrent treatment with fluralaner and emodepsid-praziquantel (treatment group). In the treatment group transient erythema was observed in two cats at the emodepsid-praziquantel administration site (two days after the 3^rd^ treatment occasion; and one day after the 5^th^ treatment occasion), one cat had a small amount of saliva in the cage four hours after the 1^st^ treatment occasion and one cat had vomited two days after the 5^th^ treatment occasion. In the control group one cat had vomited two days after the 3^rd^ treatment occasion and another cat one day after the 4^th^ treatment occasion. All of these observations were mild and transient and none required treatment or affected the general health condition of the cats. These observations are consistent with the potential adverse events specified on the individual product leaflets. There were no findings suggesting an increased safety risk associated with the concurrent treatment of cats with fluralaner and emodepsid-praziquantel.

Other clinical observations, which included one case of sporadic coughing, one case of sporadic sneezing and one case of mild skin irritation on the chin in the treatment group, are considered to be findings commonly observed in a cat colony. These findings were also minor, transient and none required treatment or affected the general health condition of the cats.

There were no obvious changes in bodyweights during the study (Table 1), and the statistical analysis using mixed models analysis of variance for a repeated measures design did not reveal any statistically significant differences between groups (*P* = 0.7070).

No unexpected clinical observations were observed over this extended study period, with multiple consecutive treatments with fluralaner and an emodepsid-praziquantel combination. The study results support the safe concurrent use of these products and are consistent with previous data showing no interactions between fluralaner and other routinely used veterinary medicinal products in cats [1] or dogs [4–8].

## Conclusion

Concurrent treatment with fluralaner, emodepsid and praziquantel is well tolerated in cats.

## References

[1] European Commission. Community register of veterinary medicinal products, Product information, Annex 1 Summary of product characteristics Bravecto spot-on solution for cats. 2016. http://ec.europa.eu/health/documents/community-register/html/v158.htm.

[2] Kilp S, Ramirez D, Allan MJ, Roepke RKA. Comparative pharmacokinetics of fluralaner in dogs and cats following topical or single intravenous administration. Parasit Vectors. in press. 10.1186/s13071-016-1564-8 PMC4886404 27241240

[3] European Commission (2010). Community register of veterinary medicinal products, Product information, Annex 1 Summary of product characteristics Profender.

[4] European Commission (2014). Community register of veterinary medicinal products, Product information, Annex 1 Summary of product characteristics Bravecto chewable tablets for dogs.

[5] European Commission. Community register of veterinary medicinal products, Product information, Annex 1 Summary of product characteristics Bravecto spot-on solution for dogs. 2016. http://ec.europa.eu/health/documents/community-register/html/v158.htm

[6] Walther FM, Fisara P, Allan MJ, Roepke RKA, Nuernberger MC (2014). Safety of the concurrent treatment of dogs with Bravecto™ (fluralaner) and Scalibor™ protectorband (deltamethrin). Parasit Vectors.

[7] Walther FM, Fisara P, Allan MJ, Roepke RKA, Nuernberger MC (2014). Safety of concurrent treatment of dogs with fluralaner (Bravecto™) and milbemycin oxime – praziquantel. Parasit Vectors.

[8] Walther FM, Allan MJ, Roepke RKA (2015). Plasma pharmacokinetic profile of ivermectin and fluralaner (Bravecto™) following concurrent administration to dogs. Parasit Vectors.

