# Erratum to: Safety of concurrent treatment of cats with fluralaner and emodepsid-praziquantel

**DOI:** 10.1186/s13071-016-1652-9

**Published:** 2016-06-28

**Authors:** Feli M. Walther, Mark J. Allan, Rainer K. A. Roepke

**Affiliations:** Merck Animal Health, 2 Giralda Farms, Madison, NJ USA; MSD Animal Health Innovation GmbH, Zur Propstei, 55270 Schwabenheim, Germany

## Erratum

Following publication of the original article [1], an error in the spelling of the active ingredient emodepside was noted. The correct spelling is emodepside and not emodepsid. This applies to the entire article, including the title.
